# Genetic landscape and personalized tracking of tumor mutations in Vietnamese women with breast cancer

**DOI:** 10.1002/1878-0261.13356

**Published:** 2023-01-15

**Authors:** Van‐Anh Nguyen Hoang, Sao Trung Nguyen, Trieu Vu Nguyen, Thanh Huyen Pham, Phuoc Loc Doan, Ngoc Thanh Nguyen Thi, Minh Long Nguyen, Thi Cuc Dinh, Dinh Hoang Pham, Ngoc Mai Nguyen, Duy Sinh Nguyen, Du Quyen Nguyen, Y‐Thanh Lu, Thanh Thuy Thi Do, Dinh Kiet Truong, Minh‐Duy Phan, Hoai‐Nghia Nguyen, Hoa Giang, Lan N. Tu

**Affiliations:** ^1^ Medical Genetics Institute Ho Chi Minh City Vietnam; ^2^ Gene Solutions Ho Chi Minh City Vietnam; ^3^ MEDIC Medical Center Ho Chi Minh City Vietnam; ^4^ Thu Duc City Hospital Ho Chi Minh City Vietnam; ^5^ Department of Oncology, Faculty of Medicine Nguyen Tat Thanh University Ho Chi Minh City Vietnam; ^6^ University of Medicine and Pharmacy Ho Chi Minh City Vietnam

**Keywords:** circulating tumor DNA, minimal residual disease, mutational landscape, next‐generation sequencing, somatic mutation

## Abstract

Breast cancer is the leading cause of cancer death in Vietnamese women, but its mutational landscape and actionable alterations for targeted therapies remain unknown. After treatment, a sensitive biomarker to complement conventional imaging to monitor patients is also lacking. In this prospective multi‐center study, 134 early‐stage breast cancer patients eligible for curative‐intent surgery were recruited. Genomic DNA from tumor tissues and paired white blood cells were sequenced to profile all tumor‐derived mutations in 95 cancer‐associated genes. Our bioinformatic algorithm was then utilized to identify top mutations for individual patients. Serial plasma samples were collected before surgery and at scheduled visits after surgery. Personalized assay tracking the selected mutations were performed to detect circulating tumor DNA (ctDNA) in the plasma. We found that the mutational landscape of the Vietnamese was largely similar to other Asian cohorts, showing higher *TP53* mutation frequency than in Caucasians. Alterations in *PIK3CA* and PI3K signaling were dominant, particularly in our triple‐negative subgroup. Using top‐ranked mutations, we detected ctDNA in pre‐operative plasma in 24.6–43.5% of the hormone‐receptor‐positive groups and 76.9–80.8% of the hormone‐receptor‐negative groups. The detection rate was associated with breast cancer subtypes and clinicopathological features that increased the risk of relapse. Interim analysis after a 15‐month follow‐up revealed post‐operative detection of ctDNA in all three patients that had recurrence, with a lead time of 7–13 months ahead of clinical diagnosis. Our personalized assay is streamlined and affordable with promising clinical utility in residual cancer surveillance. We also generated the first somatic variant dataset for Vietnamese breast cancer women that could lay the foundation for precision cancer medicine in Vietnam.

## Graphical abstract

The authors determine, for the first time, the somatic variant landscape of Vietnamese women with breast cancer, and establish a personalized tumor‐informed assay (K‐Track^®^) to detect ctDNA in liquid biopsy. This assay is streamlined and affordable, with promising clinical utilities in both residual cancer surveillance and actionable mutation profiling for breast cancer patients.

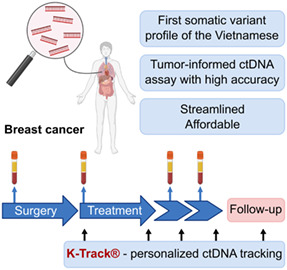

**Abbreviation**
BC: breast cancer
CA 15‐3: cancer antigen 15‐3
cfDNA: cell‐free DNA
CHIP: clonal hematopoiesis of indeterminate potential
COSMIC: catalog of somatic mutations in cancer
ctDNA: circulating tumor DNA
ER: estrogen receptor
FFPE: formalin‐fixed paraffin‐embedded
GATK: Genome Analysis Tool Kit
gnomAD: Genome Aggregation Database
HER2: human epidermal growth factor receptor 2
HR: hormone receptors
IHC: immunohistochemical
LOD: limit of detection
MAF: mutation annotation file
mPCR: multiplex polymerase chain reaction
NGS: next generation sequencing
PR: progesterone receptor
VAF: variant allele frequency
WBC: white blood cells

## 1 Introduction

Breast cancer is the most common cancer and the leading cause of cancer death in women worldwide [1]. In Vietnam, it accounts for 25.8% of all cancer cases in women, with 22 000 new cases and 9000 deaths in 2020 [1]. Recent advances in next‐generation sequencing (NGS) have shed light on genomic alterations underlying breast cancer subtypes and paved the way for successful development of targeted therapies such as PIQRAY^®^ (alpelisib) for certain *PIK3CA* mutations [2]. In developing countries like Vietnam, however, access to diagnostic genetic testing is still limited due to high cost and lack of trained laboratories and personnel. Therefore, there are currently no data about the mutational spectrum of breast cancer in Vietnam and the translational potential for precision medicine remains unknown.

Despite improved breast cancer prognosis over the last decade, the 5‐year survival probability of Vietnamese breast cancer women was reported to be 74%, lower than other Asian countries with the same stage distribution at diagnosis [3]. One of the main causes of cancer death was metastatic recurrence, which could be attributed to residual cancer cells remaining after curative‐intent treatment including surgery and adjuvant therapies. Conventional methods to monitor patients are imaging and blood tests to detect protein biomarkers such as CA 15‐3, both of which have limited sensitivity and specificity to detect residual tumor burden and hence often fail to identify patients at risk for relapse early [4, 5].

Circulating tumor DNA (ctDNA), a type of cell‐free DNA (cfDNA) released from cancer cells into the bloodstream, has emerged as a new potential biomarker to monitor treatment response. ctDNA can be distinguished from normal cfDNA based on different somatic alterations such as single‐nucleotide variant mutations. Recent scientific evidence shows that residual tumor monitoring by ctDNA in liquid biopsy is effective for solid tumors including breast cancer. Patients who were positive for ctDNA after treatment had a significantly higher risk of recurrence and metastasis compared to those negative for ctDNA [4, 6]. In addition to its prognostic value, ctDNA monitoring allowed detection of breast cancer relapse earlier than conventional methods by an average lead time of 8.9–10 months and up to 2 years [4, 6]. Such time window would allow for opportune intervention and improve patient outcomes. Currently, this sophisticated ctDNA monitoring technology is only available in developed countries and remains unaffordable for majority of the patients.

With the goal of making personalized and precision medicine accessible and affordable to the Vietnamese, we established K‐Track^®^, a tumor‐informed liquid biopsy assay to detect ctDNA in breast cancer patients to monitor treatment response. Our interim analysis showed that the assay could stratify patients based on post‐treatment ctDNA status and detect relapse early ahead of clinical diagnosis. Besides that, we also profiled for the first time the somatic mutation spectrum of Vietnamese women with breast cancer, which has translational potential for both current and future targeted therapies.

## 2 Materials and methods

### 2.1 Patients and sample collection

**Fig. 1:**
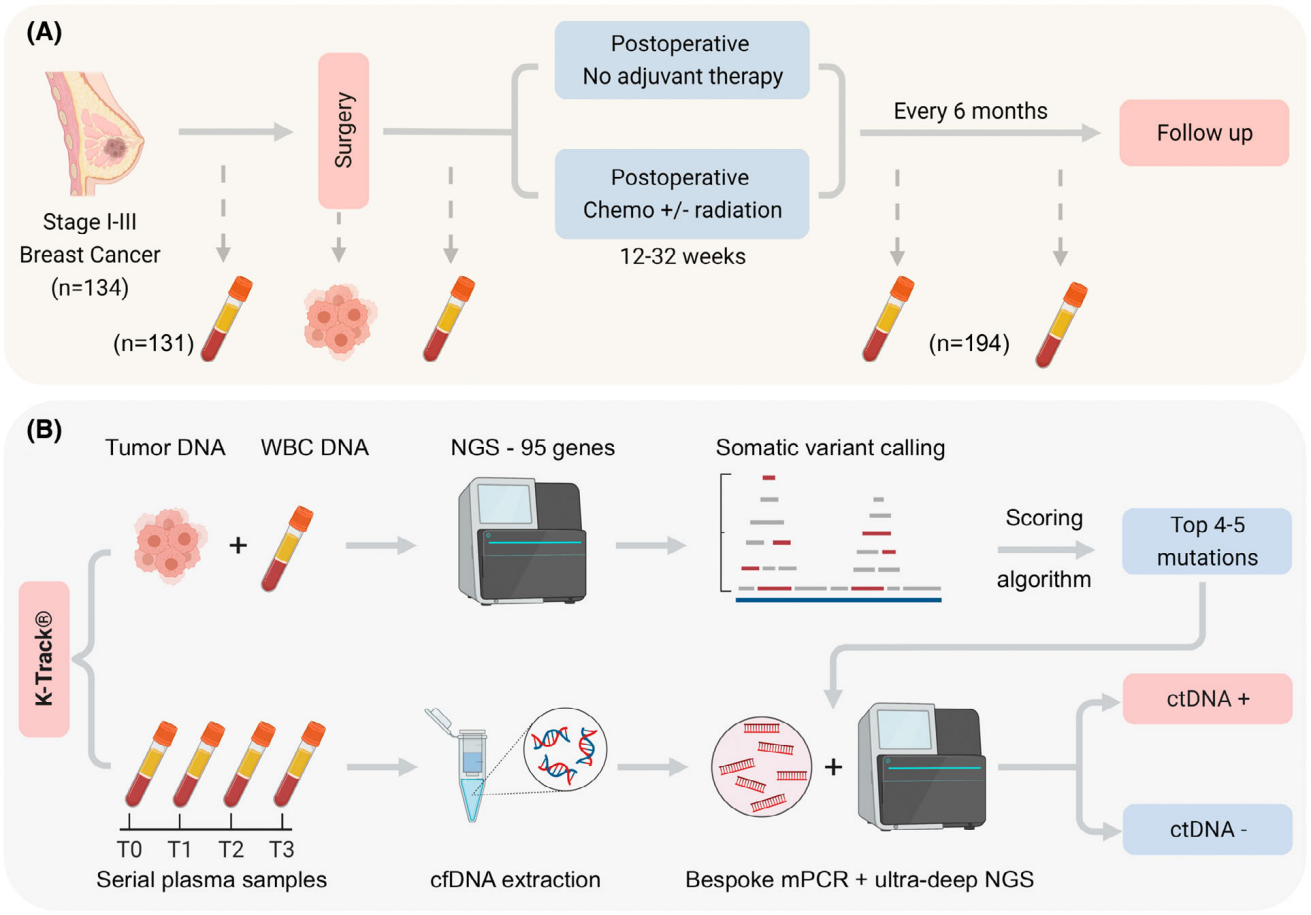
Schematic of study design and K‐track® assay. (A) 134 patients with primary breast cancer stage I‐III, eligible for curative‐intent surgery were enrolled. Serial plasma samples were collected before surgery and after surgery at scheduled visits. Formalin‐fixed paraffin‐embedded (FFPE) samples of surgically removed tumors were also collected. Patients were followed‐up to record clinical outcomes. (B) Paired FFPE and white blood cells (WBC) DNA of the same individual were sequenced to identify tumor‐specific somatic mutations in 95 cancer‐associated genes. Top 4–5 mutations were selected by our scoring algorithm and tracked in plasma samples using a bespoke multiplex polymerase chain reaction (mPCR) assay and ultra‐deep next‐generation sequencing (NGS).

One hundred and thirty‐four patients who were at least 18 years old, diagnosed with stage I–III breast cancer and eligible for curative‐intent surgery were recruited from March 2021 to May 2022 in MEDIC Medical Center and Thu Duc City Hospital, Ho Chi Minh city, Vietnam. Patients who had received prior cancer treatment, or had recurrence, metastasis or multiple malignancies in the past 5 years prior to the time of study entry were excluded. All patients received treatment according to standard‐of‐care with the treating surgical and medical oncology teams. Ten microliters of peripheral blood was serially collected before surgery and at scheduled follow‐up visits after surgery. Formalin‐fixed paraffin‐embedded (FFPE) samples of surgically removed tumors and the immunohistochemical (IHC) staining results for hormone receptors (HR): estrogen receptor (ER) and progesterone receptor (PR) and human epidermal growth factor receptor 2 (HER2) were provided by pathologists. Breast tumor specimens were HR‐positive if at least 1% of tumor nuclei were stained positive for ER, PR or both. HER2 was defined positive by an immunohistochemistry score of 2+ and 3+. FFPE sections containing at least 60% tumor cellularity were used for genomic analysis. Clinicopathological information and medical history were provided by physicians in a standardized electronic format. The date of clinical recurrence was the date of imaging or biopsy confirming recurrence and/or metastasis. Patient demographics are listed in Table S1; study design and workflow are illustrated in Fig. 1 (created with BioRender.com).

In all analysis, patients were divided into four groups according to the IHC status of their tumor: HR+ HER2−, HR+ HER2+, HR− HER2+, HR− HER2−. In one analysis, patients in the HR+ groups were further classified into low‐ and high‐risk of relapse based on clinicopathological features established in previous studies [7, 8]. A clinically high‐risk breast cancer patient must meet one of the following criteria: (a) at least a 15% predicted risk of death within 10 years using epredict V2.1 (https://breast.predict.nhs.uk/tool), or (b) tumor size > 5 cm (T3) regardless of lymph node status, or (c) *N* ≥ 4 lymph nodes, or (d) *N* = 1–3 lymph nodes and at least one of the following: tumor size > 3 cm, or high histological grade 3, or high genomic risk defined as Oncotype Dx Recurrence Score > 26. FFPE and blood samples were subjected to the K‐Track^®^ assay workflow as illustrated in Fig. 1 and described in detail below.

The study methodologies conformed to the standards set by the Declaration of Helsinki. All patients provided written informed consent to participate in the study and to the anonymous use of their samples, clinical and genomic data for this study. All genomic data were de‐identified and aggregated for the genetic analysis of the cohort. The study was approved by the institutional ethics committees of the University of Medicine and Pharmacy, Ho Chi Minh City (#300/HDDD) and Thu Duc city Hospital (#17/HDDD).

### 2.2 Tumor sample processing

Genomic DNA was isolated from FFPE samples by the QIAamp DNA FFPE Tissue Kit (Qiagen, Qiagen, MA, USA) according to manufacturer's instructions. Matching genomic DNA from white blood cells (WBC) of the same individual was extracted from the buffy coat by MagMAX™ DNA Multi‐Sample Ultra 2.0 kit (ThermoFisher, Waltham, MA, USA) according to manufacturer's instructions. DNA fragmentation and library preparation for paired FFPE and WBC samples were performed using the NEBNext Ultra II FS DNA library prep kit (New England Biolabs, Ipswich, MA, USA) following the manufacturer's instructions. Libraries were pooled together and hybridized with predesigned probes for 95 targeted genes (Integrated DNA Technologies, Coralville, IA, USA). This gene panel includes the top 20 most frequently mutated genes in all breast cancer subtypes and other solid tumors as reported in the Catalog of Somatic Mutations in Cancer (COSMIC) database (Table S2). Massive parallel sequencing of DNA libraries was performed on the DNBSEQ‐G400 sequencer (MGI, Shenzhen, China) with the average target coverage of 200× (range 89–308×).

### 2.3 Tumor variant calling and ranking

Sequencing data were processed based on best practices workflows from Genome Analysis Tool Kit (gatk) for somatic variant calling [9]. Specifically, reads were aligned to the human reference genome (GRCh38) by bwa‐mem (v0.7.15) [10]. picard (v2.25.6) [11] was then used for post‐alignment procedures including sorting, marking duplicated reads and assessing alignment quality. Somatic variants were called by gatk mutect2 (v4.0.12.0) [12] in the tumor‐normal mode for paired FFPE and WBC samples using: (a) a panel of normals retaining sites presented in at least two samples from in‐house normal pool (146 samples); (b) the population allele frequency from The Genome Aggregation Database (gnomAD), to remove sequencing noise and germline variants. The set of called variants were further characterized by assessing their functional impact using variant effect predictor (v105) with the data from COSMIC (v95), Clinvar (v20220103) and OncoKB database [13] (Table S3). The annotated Variant Call Format was then converted to the Mutation Annotation File (MAF) format using vcf2maf (v1.6.21; 10.5281/zenodo.593251). The MAF data were analyzed and visualized using the ‘maftools’ in r package v3.4.2 [14].

All non‐synonymous mutations were ranked by our scoring algorithm to identify the most potential tumor‐derived driver and clonal mutations to track. Ranking criteria for mutations include being (a) predicted to be pathogenic/deleterious in the Clinvar and COSMIC databases or by SIFT and Polyphen programs; (b) a stop‐gained mutation in a tumor suppressor gene (by COSMIC classification); (c) a mutation in an oncogene (by COSMIC classification) and reported more than three times in COSMIC database; (d) validated as a tumor‐derived mutation according to our in‐house genetic database. Variant allele frequency (VAF) in FFPE was considered as a separate criterion to prioritize ranked mutations. Exclusion criteria included mutations being located in high GC or low complexity regions. The final top mutations unique to each patient were selected to design bespoke multiplex PCR assays on cfDNA.

### 2.4 Plasma sample processing and multiplex PCR

Blood samples were collected in the BD Vacutainer K2 EDTA tubes (BD, Franklin Lakes, NJ, USA), stored at 4 °C and processed within 6 h. Tubes were centrifuged at 4 °C, 2000 ***g*** × 10 min; the plasma layer was further centrifuged at 4 °C, 16 000 ***g*** × 10 min. cfDNA was then extracted from clean plasma fractions using the MagMAX™ Cell‐Free DNA Isolation Kit (ThermoFisher) according to the manufacturer's instructions. cfDNA concentration was quantified using the QuantiFluor^®^ dsDNA system (Promega, Madison, WI, USA). A concentration of ≥ 0.1 ng/μL or a total of ≥3 ng cfDNA was required for downstream analysis. Compatible pairs of primers were designed for each patient by primer3plus software and synthesized by PhuSa Biochem (Ho Chi Minh city, Vietnam). An average cfDNA input for mPCR assay was 5.4 ng (range 3–30 ng). cfDNA fragments carrying the selected mutation sites were amplified in a multiplex PCR (mPCR) reaction containing designed primer pairs and enzyme KAPA HiFi DNA Polymerase (Roche, Roche Sequencing Solutions, Indianapolis, IN, USA). Amplified cfDNA fragments were ligated with indexes and adaptors in a second PCR reaction, and then sequenced on the NextSeq 2000 system (Illumina, San Diego, CA, USA) with an average depth of > 100 000× per amplicon. Amplicons with less than 10 000× coverage were considered failed.

### 2.5 Plasma variant calling and ctDNA analysis

The raw fastq data of amplicons were first removed adapters with trimmomatic (v0.39) [15], then mapped to the human reference genome (GRCh38) using bwa‐mem (v0.7.15), sorted and marked duplicates using picard (v2.25.6). Alignment quality metrics were obtained using picard's collecthsmetrics. Variant calling was performed using mpileup from samtools (v1.11) [16].

To determine limit of detection (LOD), we used commercial mutation reference standards Tru‐Q1 and Tru‐Q0 (Horizon Discovery, Cambridge, UK) and titrate the somatic mutations at average VAFs (based on DNA input) of 3%, 0.5%, 0.1%, 0.05% and 0%. The mixtures were fragmented with enzyme NEBNext DNA fragmentase (New England Biolabs) to mimic cfDNA length and then processed through the mPCR workflow as above. The observed VAF was compared with the expected VAF for each mutation to determine the LOD of the assay. Additionally, negative cfDNA samples isolated from 100 healthy human plasmas were also subject to the same workflow to determine the false‐positive rate of the assay.

A sample was called positive for ctDNA if at least one mutation was detected with VAF ≥ selected LOD. Mean VAF of a sample was calculated as mean of all positive mutations detected. If none of the mutations was positive, mean VAF was calculated as mean of all mutations.

### 2.6 Statistical analysis

For continuous variables including number of mutations, VAF, cfDNA and ctDNA levels, Mann–Whitney *U* test was performed for comparison between two groups and Kruskal–Wallis with *post hoc* Dunn's test was performed for comparison among more than two groups. For categorical variables including the mutation frequency and detection rate, Chi‐squared test and Fisher's exact test were used. All statistical tests were performed in graphpad prism (san diego, ca, usa) and considered significant at *P* < 0.05.

## 3 Results

### 3.1 Study cohort and design

Our cohort of 134 breast cancer women had a median age of 52% and 53.0% were postmenopausal. Based on the IHC staining, patients were divided into four groups: HR+ HER2− (51.5%), HR+ HER2+ (17.2%), HR− HER2+ (19.4%) and HR− HER2− (11.2%; Table S1). In the two HR+ groups, we further classified patients into groups with low and high risk of relapse by the clinicopathological criteria described in the Materials and methods. All patients had non‐metastatic carcinoma at TNM stage I (23.1%), II (51.5%) and III (22.4%); majority had 1 tumor with an average tumor size of 2.4 cm. 68.7% of the tumors had intermediate histological grade, and 58.2% of the cases had spread to lymph nodes (Table S1).

In our K‐Track^®^ assay, FFPE tumor and serial plasma samples were collected before and after surgery at scheduled visits (Fig. 1A). DNA from paired FFPE and WBC were hybridized to predesigned 95‐gene panel to identify tumor‐derived mutations. Our developed scoring algorithm ranked and selected on average the top 4–5 mutations for each patient, which were then used to track ctDNA in a bespoke mPCR assay. The detection of ctDNA in the plasma was compared with clinical outcomes (Fig. 1B).

### 3.2 Mutational landscape of different BC subtypes

**Fig. 2:**
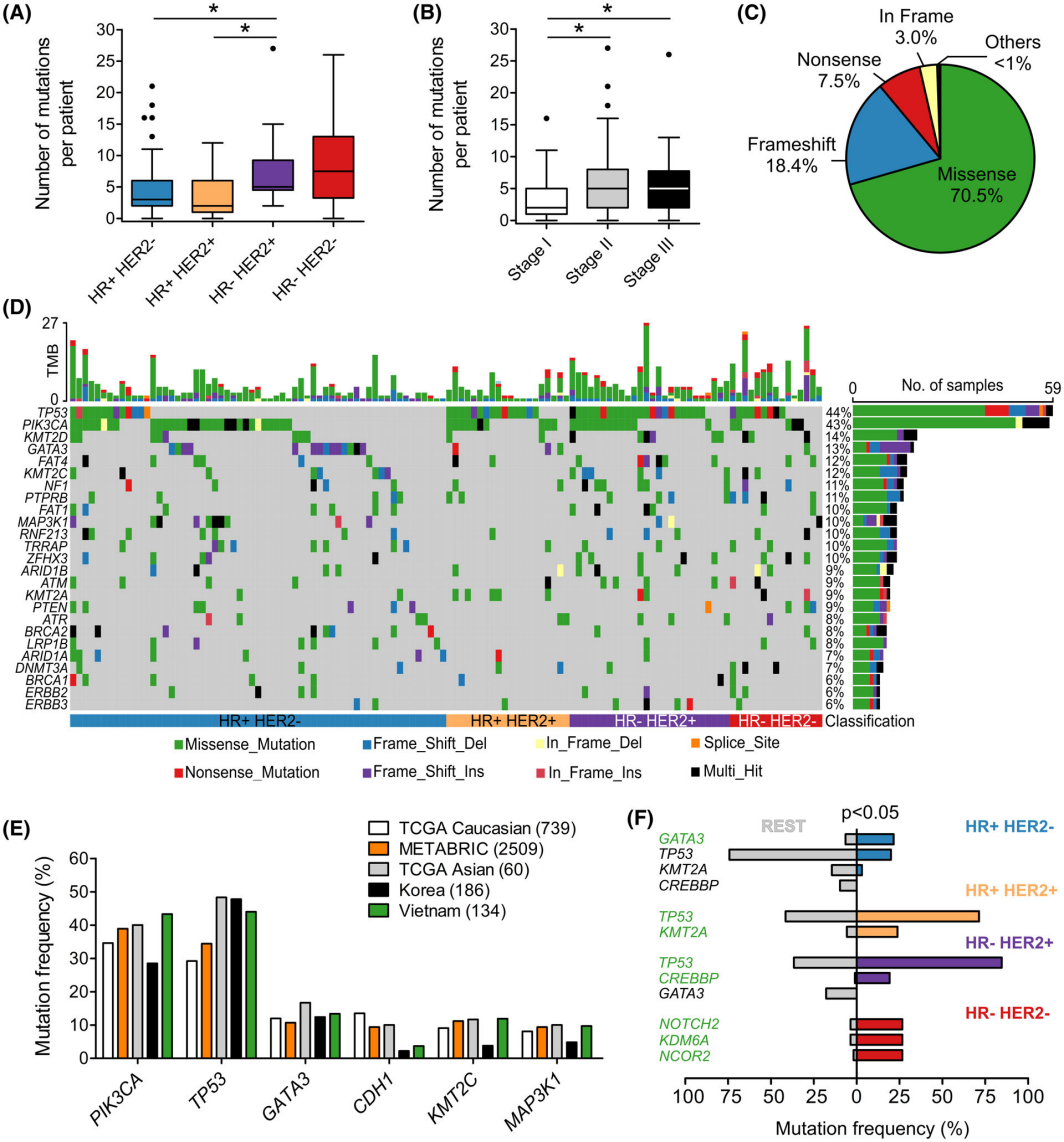
Mutational spectrum of 95 genes in Vietnamese breast cancer women. (A) The number of somatic mutations identified per patient was significantly higher in the HR− HER2+ compared to the HR+ groups. (B) The mutation burden was significantly lower in stage I compared to stage II and III. (C) Pie chart showing the distribution of mutation classes identified in 95 genes. (D) The top 25 significantly mutated genes in the cohort. (E) Frequency of top highly mutated genes in breast cancer was compared between our cohort and published datasets of Caucasian and Asian cohorts. (F) Pairwise analysis identified oncogenic alternations significantly associated with different subtypes. Genes in green color were significantly more altered in a particular group compared to the rest while genes in black color were significantly less altered. HR, Hormone receptors; HER2, Human epidermal growth factor receptor 2. *P < 0.05; Kruskal–Wallis and *post hoc* Dunn's test for (A) and (B); Fisher's exact test for (F). (A), (B): Boxplots with Tukey whiskers.

Sequencing results of paired FFPE‐WBC showed an average of 5, 4, 7 and 9 mutations per patient for HR+ HER2−, HR+ HER2+, HR− HER2+ and HR− HER2− subgroups, respectively (Fig. 2A). The number of somatic mutations identified for the HR+ groups was significantly lower than the HR− HER2+ group. The mutational burden was significantly lower in tumors at stage I compared to stage II and III (Fig. 2B). Majority of mutations were missense (70.5%), followed by frameshift (18.4%) and nonsense (7.5%) mutations (Fig. 2C).

The most frequently mutated genes in our cohort were *TP53* (44.0%) and *PIK3CA* (43.3%; Fig. 2D). We compared this data to published breast cancer datasets from the Caucasian cohorts: TCGA Caucasian (*n* = 739) [17] and METABRIC (*n* = 2509) [18]; as well as the Asian cohorts: TCGA Asian (*n* = 60) [17] and the Korean (*n* = 186) [19]. This Korean cohort was enriched with younger patients but the top mutated genes were shown similar to a general Korean cohort [20]. Overall, the mutation rate of *TP53* in the Vietnamese cohort was higher than the Caucasian but similar to the Asian cohorts (Fig. 2E). The *CDH1* mutation rate was lower than the Caucasian but similar to the Korean (Fig. 2E).

Mutational analysis across four subtypes identified *TP53* as the top mutated gene in the HR+ HER2+ (65.2%), HR− HER2+ (84.6%) and HR− HER2− (56.3%) while *PIK3CA* was the most mutated gene in the HR+ HER2− (44.9%; Fig. S1). The mutational landscapes in the HR+ groups were fairly similar to other breast cancer cohorts (Fig. S2). The HR− HER2− group was more distinctive that we observed much higher frequency of mutations in *PIK3CA* but lower frequency in *TP53* compared to both other Caucasian and Asian cohorts (Fig. S2). We then performed pairwise analysis to identify the mutation signature that distinguished each breast cancer subtype from the rest (Fig. 2F). The HR+ HER2− group had significantly more mutations in *GATA3* while *TP53* and *KMT2A* mutations were signature of the HR+ HER2+ group (*P* < 0.05). *TP53*, *CREBBP* mutations were altered more in the HR− HER2+; and the HR− HER2− tumors were more frequently mutated in *NOTCH2*, *KDM6A* and *NCOR2* compared to others (*P* < 0.05; Fig. 2F). Among the 95 examined genes, *PIK3CA* showed a prominent mutation hotspot at amino acid Histidine 1047, as H1047R/L accounted for 56.1% of all *PIK3CA* mutated cases (Fig. S3). We also compared the frequency of the most common mutations in *PIK3CA*, *TP53*, *GATA3* and *AKT1* genes among our cohort and others (Table S4). *PIK3CA* H1047R/L (27.2%) was found more prevalent in the Vietnamese than all other cohorts (9.8–19.1%). The list of all identified variants is provided in Table S5.

### 3.3 Oncogenic signaling pathways and clinical actionability

**Fig. 3:**
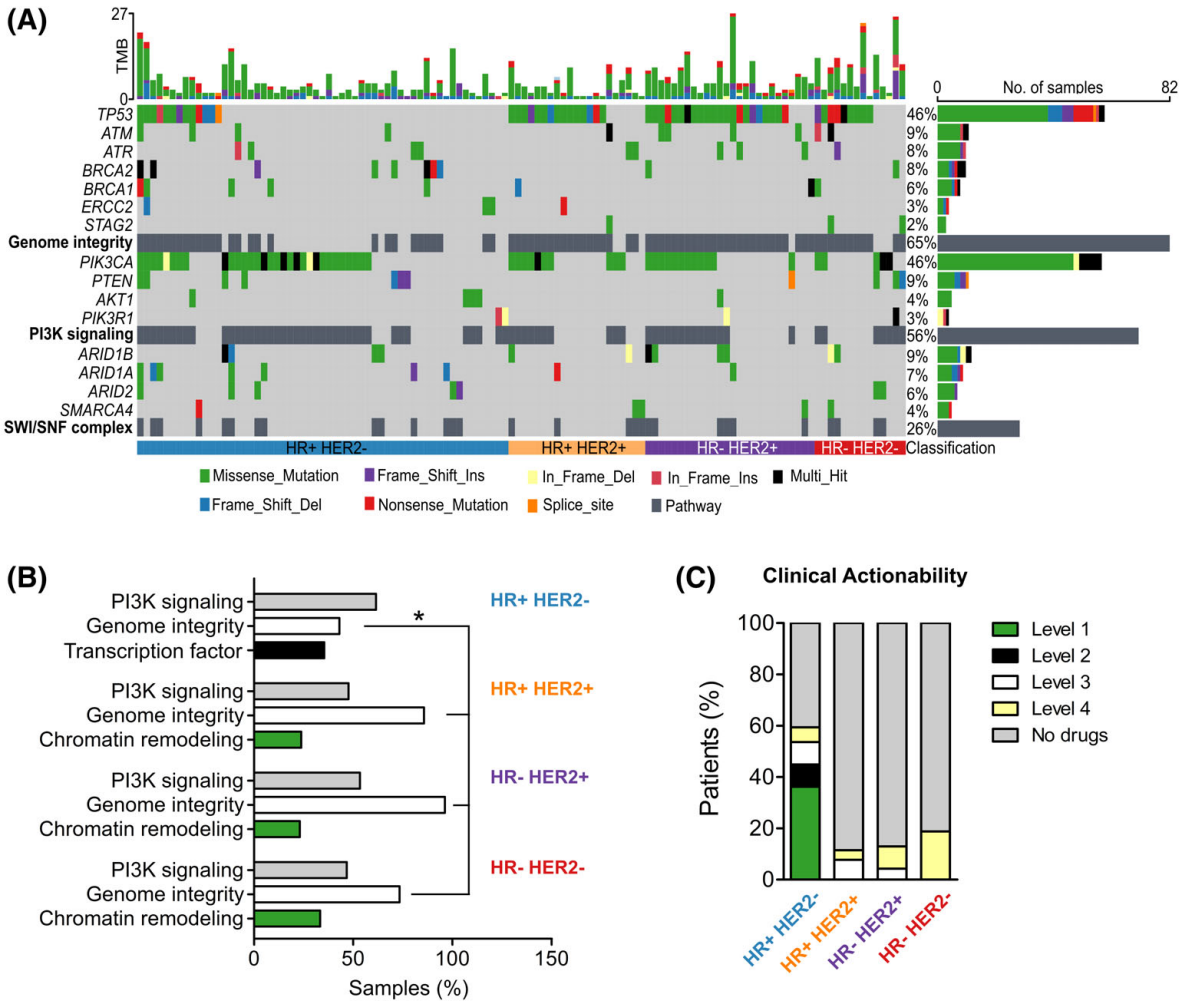
Oncogenic signaling pathways and actionable alterations in breast cancer subtypes. (A) The top three signaling pathways with frequent oncogenic alterations in our cohort were genome integrity, PI3K signaling and chromatin SWI/SNF remodeling complex. (B) The top three altered signaling pathways were different among breast cancer subtypes. HR+ HER2− had significantly less alterations in genome integrity pathway compared to other groups. Transcription factor pathway was highly mutated specifically in the HR+ HER2− group. (C) Proportion of patients in each subtype that had actionable alterations predictive of treatment response to a drug at different levels of evidence stratified by OncoKB database. HR, Hormone receptors; HER2, Human epidermal growth factor receptor 2. *P < 0.05; Fisher's exact test for (B).

The top three signaling pathways being altered in our breast cancer cohort were genome integrity (*TP53*, *ATR*, *ATM*, *BRCA1/2*), Phosphoinositide 3‐kinase PI3K signaling (*PIK3CA*, *PTEN*, *AKT1*) and Switch/Sucrose non‐fermentable SWI/SNF chromatin remodeling complex (*ARID1A*, *ARID2*, *SMARCA4*) with the mutation frequency of 64.6%, 55.9% and 26.0%, respectively (Fig. 3A). Analysis across breast cancer subtypes found that HR+ HER2− group had significantly less alteration in genome integrity pathway compared to other groups (*P* < 0.05). Particularly, the transcription factor pathway (*GATA3*, *ZFHX3*, *FOXA1*) was highly mutated specifically in the HR+ HER2− group (Fig. 3B).

We further characterized actionable targets in our cohort who might benefit from genetic sequencing for targeted therapies. The OncoKB database [13], an expert‐curated precision oncology knowledge base, was used to classify somatic alterations with treatment implications stratified by different levels of evidence [13]. The list of alterations and corresponding drugs for breast cancer were listed in Table S3. In total, we found that 59.4% HR+ HER2− patients had at least 1 mutation that could be targeted by a drug; majority (36.2%) were *PIK3CA* mutations classified as level 1 biomarkers for FDA‐approved drug Alpelisib (Fig. 3C, Table S3). Other breast cancer subtypes had mutations being targeted by drugs in clinical trials (level 3) or only demonstrated in biological research (level 4; Fig. 3C, Table S3).

### 3.4 Tracking ctDNA to monitor treatment response

**Fig. 4:**
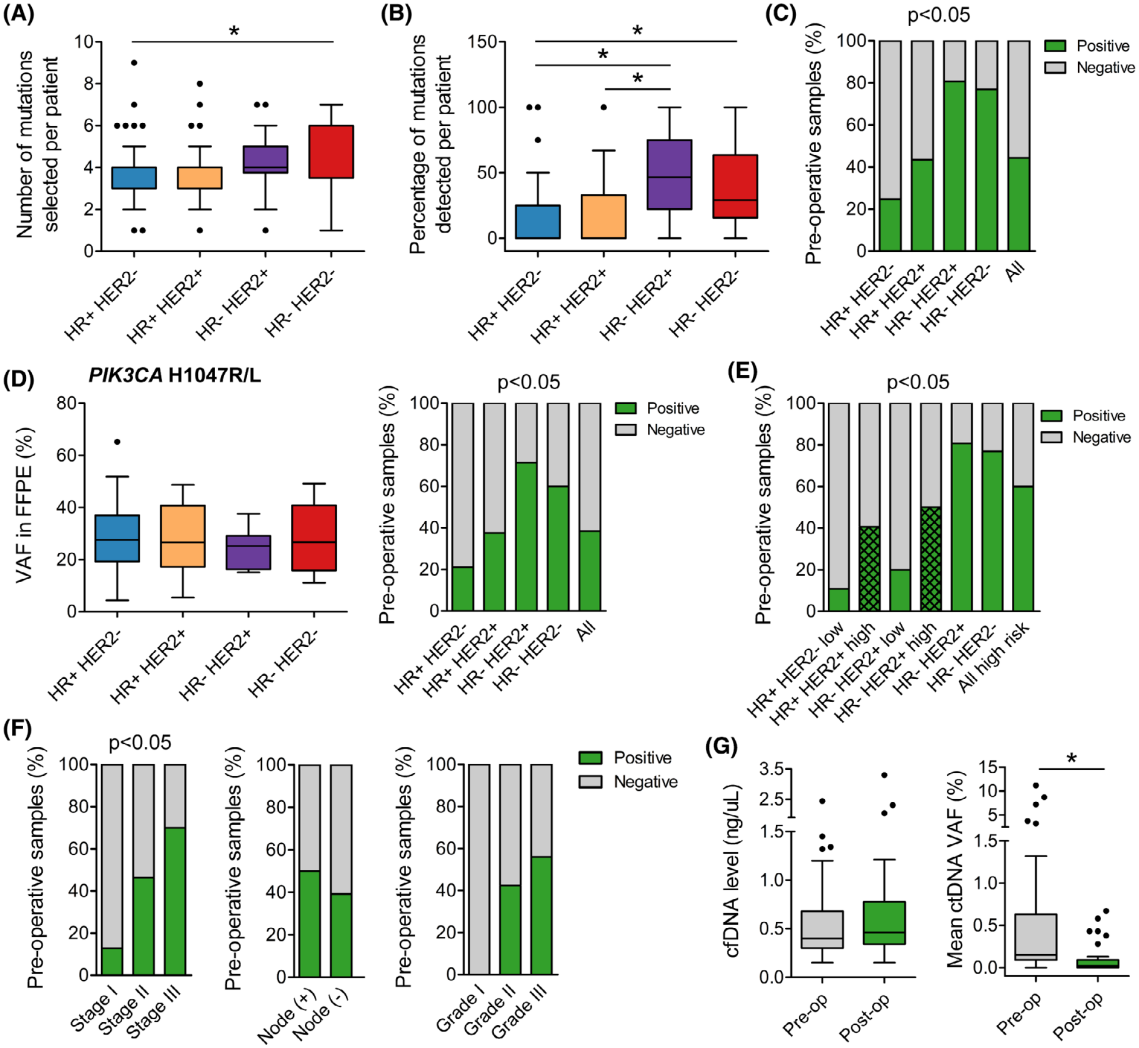
Analysis of circulating tumor DNA (ctDNA) in pre‐operative plasma samples. (A) The number of mutations selected to track in each patient was significantly lower in the HR+ HER2− group than in the HR− HER2− group. (B) The percentage of tracked mutations that could be detected in the plasma was significantly lower in the HR+ groups compared to HR‐ groups. (C) Pre‐operative detection rate was associated with breast cancer subtypes and was significantly lower in the HR+ groups compared to HR− groups. (D) The detection rate of *PIK3CA* H1047R/L in the plasma followed the exact same trend as the overall detection rate even though the mutation was identified at similar variant allele frequency (VAF) in tumors across subtypes. (E) When HR+ groups were stratified by clinicopathological features that increase risk of relapse, the detection rate was significantly lower in the low‐risk than the high‐risk group of HR+ HER2−. (F) Detection was associated with TNM stage as the detection rate in stage I was significantly lower than in stage II and III. Nodal and grade status were not found to affect pre‐operative detection rate. (G) Levels of pre‐operative and post‐operative cell‐free DNA (cfDNA) were not different while VAF of ctDNA significantly reduced after surgery. HR, Hormone receptors; HER2, Human epidermal growth factor receptor 2. *P < 0.05; Kruskal–Wallis and *post hoc* Dunn's test for (A), (B), (D); Mann–Whitney *U* test for (G); Chi‐squared test and Fisher's exact test for (C–F). (A), (B), (D), (G): Boxplots with Tukey whiskers.

From the list of mutations identified in the tumor, we applied an algorithm to score the mutations based on several criteria (described in Materials and methods) to determine the tumor‐derived driver and clonal mutations. Those with the highest score and highest VAF in FFPE were selected for individual patient. Based on our analysis, VAF in FFPE was the more determining factor for the likelihood of detection in plasma as mutations with VAF < 10% albeit high score were unlikely to be detected compared to those with VAF ≥ 10% (Fig. S4A). On average, we selected 4 (range 1–9) mutations per patient and the number selected was the lowest in the HR+ HER2− due to lower mutational burden (Fig. 4A).

Bespoke multiplex PCR and ultra‐deep sequencing were performed to detect ctDNA in plasma samples with an average depth of 100 000× per target. 100% of the samples had sufficient cfDNA for mPCR assay; 6.2% amplicons with less than 10 000× coverage were considered failed and removed from analysis (Fig. S4B). The assay could detect mutations at frequency below 0.05% but it also recorded false‐positive signals from healthy plasma samples at VAF < 0.05% (Fig. S4C). Therefore, we chose the cut‐off of 0.05% to keep the false‐positive rate below 1% (Fig. S4D). Any mutation with VAF ≥ 0.05% in plasma samples was called ‘detected’ or ‘positive’.

The average number of positive mutations detected per patient was 2 (range 1–5), accounting for 21.3% of tracked mutations; there was no correlation between the VAF of a mutation in FFPE and its detected VAF in ctDNA (Fig. S4E). The percentage of positive mutations detected in the HR+ groups was significantly lower than the HR‐ groups (Fig. 4B). A plasma sample was called ‘positive’ for ctDNA when at least 1 tracked mutation was positive. The overall detection rate in pre‐operative plasma samples was 44.3% and segregated by subtypes. The detection rate was lowest in the HR+ HER2− (24.6%), followed by the HR+ HER2+ (43.5%) and highest in the HR− HER2+ (80.8%) and HR− HER2− (76.9%) groups (Fig. 4C). To examine whether such difference was truly associated with the subtypes or due to unknowing bias in the mutation selection, we compared the detection rate of a hotspot mutation *PIK3CA* H1047R/L as they were detected in tumors across all subtypes. Although the VAF of *PIK3CA* H1047R/L in FFPE samples were similar among the groups, the pre‐operative detection rate of this mutation followed the exact same trend and confirmed the poorer release of ctDNA in the HR+ groups (Fig. 4D). We then further stratified the HR+ groups into low and high risk of relapse based on criteria associated with aggressiveness of the tumors. The detection rate was indeed significantly higher in the HR+ HER2−, high‐risk (40.6%) compared to the HR+ HER2−, low‐risk (10.8%; Fig. 4E). Excluding the two low‐risk groups, the overall pre‐operative detection rate was at 59.6%. Besides that, the detection rate was found to be associated with the TNM stage. The detection rate in stage I was significantly lower than stage II and III (Fig. 4F). We did not use the TNM stage to stratify our HR+ groups because it did not segregate the ctDNA detection rates as well as the set of clinical features described in the Materials and methods (data not shown).

**Fig. 5:**
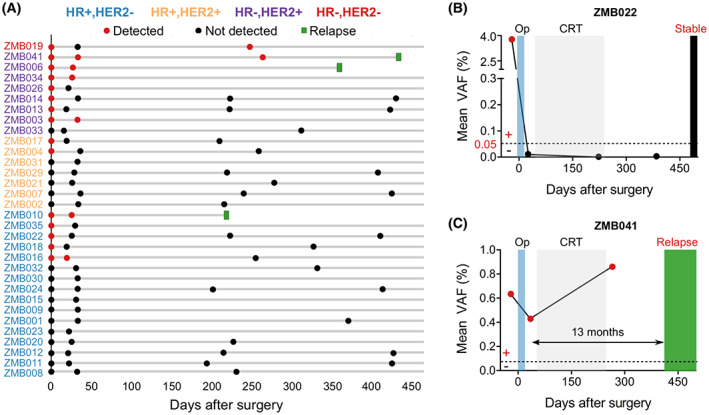
Longitudinal monitoring of circulating tumor DNA (ctDNA) and clinical outcomes. (A) Swimmer plot depicting serial ctDNA results over time and incidence of relapse or metastasis of 32 patients that had been followed up for at least 15 months. This was an interim analysis as the clinical study is ongoing. (B, C) longitudinal plot showing the mean VAF of ctDNA, treatment and clinical status over time of patients ZMB022 and ZMB041. CRT, Chemoradiotherapy; Op, Operation. Molecular relapse detection was 13 months earlier than clinically diagnosed relapse in patient ZMB041.

In patients that had positive pre‐operative ctDNA, we compared the dynamics of cfDNA and ctDNA after surgery. The results showed that total level of cfDNA was not different between pre‐op and post‐op samples. However, the ctDNA level, measured as the mean VAF of the tracked mutations, significantly reduced after surgery, correlating well with the clinical removal of tumor burden (Fig. 4G). The result of ctDNA clearance was then compared with the clinical outcomes of patients who had been followed up for at least 15 months. The average post‐op days that the ctDNA samples were collected were 155 (range 21–425). All the three patients who were clinically diagnosed with relapse and metastasis had ctDNA(+) after surgery (Fig. 5A). The lead time of ctDNA(+) detection was 7–13 months ahead of clinical diagnosis. Two case studies were illustrated in more detail: patient ZMB022 with stage II, HR+ HER2− breast cancer had undetected ctDNA in all follow‐up plasma samples and remained clinically stable (Fig. 5B); patient ZMB041 with stage III, HR− HER2+ subtype, had ctDNA(+) after surgery and the ctDNA level further increased after chemoradiation therapy, suggesting that she did not respond to adjuvant treatment but was clinically stable at that point. She later was diagnosed with liver metastasis at 14 months after surgery. This was an interim analysis of our clinical study which is ongoing and all the patients are still being followed‐up.

## 4 Discussion

In this study, we generated the first somatic variant dataset for Vietnamese patients with breast cancer using massive parallel sequencing technology. Availability of this dataset contributes to the expanding knowledge base of the genetic complexity and ethnic disparities in breast cancer. Our data showed that 94.8% of the Vietnamese patients had at least one somatic mutation in the 95 cancer‐associated genes. The HR− HER2− group showed the highest number of mutations per patient, consistent with the literature that triple‐negative breast cancer had higher tumor mutational burden than other subtypes [21, 22], making it a good candidate for immune checkpoint therapy.

Among the top mutated genes in our cohort, the frequency of *TP53* mutations, which were associated with poor prognosis [23], was higher than the Caucasian but similar to Asian cohorts [19, 24]. Prevalence of *PIK3CA* mutations (43.3%) seemed to be slightly higher, mainly due to its high frequency in our HR− HER2− group (31.3%) compared to 0–17.3% rates in other cohorts. Although this trend needs to be corroborated with a larger number of patients, the result suggested that Vietnamese triple‐negative patients might benefit from future *PIK3CA*‐targeted therapies. *CDH1* mutations, which are strongly associated with lobular breast cancer, were found less prevalent in the Vietnamese compared to the Caucasian, most likely due to the lower frequency of lobular cases at 1.5% in our cohort compared to 18.5% in the TCGA cohort [20]. Such low rate of lobular breast cancer has been reported in a different Vietnamese cohort at 5.1% [25] and in other Asian cohorts: 3.2% in the Malaysian MyBrCa cohort [20] and 3.7% in the Korean [19]. Furthermore, pairwise analysis showed that *GATA3* mutations were significantly enriched in the HR+ HER2− while *TP53* mutations were signature of HR− HER2+ group, consistent with previous data in other cohorts [17, 18]. In addition, *NOTCH2* mutations were found dominant in our HR− HER2− patients, supporting the reported role of Notch signaling in pathogenesis of triple‐negative breast cancer [24, 26].

Establishing management regimens based on precise biological processes connected to tumorigenesis is the main objective of precision cancer medicine. In this study, we identified PI3K signaling and genome integrity pathways as being the most affected in breast cancer, similar to the findings in other cohorts [18, 19, 20]. Drugs targeting candidate genes in these pathways such as *BRCA1/2* and *AKT1* are being investigated in clinical trials (Table S3). Specifically in the HR+ HER2− group, besides its hallmark PI3K signaling, we found less alterations in the genome integrity pathway but unique abundance of mutations in several transcription factors such as *GATA3*, *ZFHX3* and *FOXA1* compared to other subtypes, suggesting a distinctive pathogenesis mechanism to be investigated in the future. Furthermore, our data showed that 44.9% of the HR+ HER2− patients had mutations targeted by FDA‐approved drugs (both levels 1 and 2), mainly the PIQRAY^®^ (alpelisib) for *PIK3CA* mutations. An additional 8.7%, 7.7% and 4.3% patients in HR+ HER2−, HR+ HER2+ and HR− HER2+ groups, respectively, had mutations targeted in clinical trials. These findings give hope to future access to tailored therapy for Vietnamese breast cancer patients and also highlight the necessity of a comprehensive genetic analysis to identify actionable alterations. However, since we did not analyze *ERBB2* gene copy number, our data likely underestimated a small percentage of patients who could benefit from ERBB2‐amplification drugs but had equivocal or false negative ERBB2 IHC result.

Using tumor‐guided mutation information of the 95 genes, we designed a bespoke 5‐plex mPCR assay to detect ctDNA in serial liquid biopsy samples. This approach for K‐Track^®^ is fairly streamlined compared to studies using tumor whole exome sequencing and mPCR for 16 or more amplicons [4, 27], which could compromise the sensitivity of the assay [28]. However, a small gene panel focusing on only strong cancer‐associated genes has advantages of lower background noise, lower volume of data to process and overall lower cost of sequencing, making it more high‐throughput and affordable for routine testing in Vietnam and probably other developing countries. Despite using a small gene panel, we detected somatic mutations in 94.8% of patients that could be used for tracking. All somatic mutations were called in paired FFPE‐WBC to remove germline and Clonal hematopoiesis of indeterminate potential (CHIP) mutations as they are potential sources of false positives [29]. Furthermore, our scoring algorithm prioritized VAF and tumorigenicity of mutations based on many criteria including validation from a large in‐house genetic database of Vietnamese cancer patients, which could further reduce false positives and increase the likelihood of detection in the plasma. The analytical validation of K‐Track^®^ mPCR NGS platform allowed for the limit of detection at 0.05% and the specificity of > 99%. This LOD is lower than a few platforms that achieved LOD at 0.01% [28, 30] but comparable with several others with LOD of ≥ 0.1% [31, 32, 33].

The pre‐operative ctDNA detection rate of our K‐Track^®^ assay for all patients was 44.3%, similar to [34]; the rate in combined high‐risk groups was 59.6%, slightly lower than 63% [35] and 78% [6] detection rates in their respective high‐risk cohorts. These studies all used a much more extensive sequencing approach than K‐Track^®^ and the non‐inferiority of our result again supported both clinical and economic values of the assay. Furthermore, consistent with previous publications [6, 36], we observed that HR+ tumors seemed to release much less ctDNA into the bloodstream than the HR− tumors, posing a challenge to identify ctDNA in HR+ patients. This patient group often has good prognosis with very low rate of metastatic recurrence in the first 5 years, making the immediate monitoring of residual cancer after surgery rather unessential. However, certain clinicopathological features such as large tumor size > 5 cm, 4 or more lymph nodes involved and high Oncotype Dx score increase the relapse risk of HR+ patients to more than 10% in the first 2 years [8]. When we stratified HR+ groups based on these features, it was clear that high‐risk tumors, supposed to be more aggressive, released more ctDNA and had the ctDNA detection rate significantly higher than the low‐risk tumors. Therefore, we recommend K‐Track^®^ assay to evaluate minimal residual cancer for only high‐risk HR+ and all HR− patients due to the clinical impact and technical sensitivity.

Based on post‐operative ctDNA results, we stratified patients into two groups: ctDNA(+) and ctDNA(−) and recorded their clinical outcomes. All three cases that were clinically diagnosed with metastasis or relapse had ctDNA(+) after surgery, with the lead time of 7–13 months, comparable with the median lead time of 8.9–10 months in other assays [4, 6].

The major limitation of this report was that the clinical data were not yet mature as the study is ongoing. The results reported here were not enough to conclude the sensitivity and specificity of the K‐Track^®^ assay in predicting relapse. Besides that, the current design for K‐Track^®^ assay was tumor‐guided, making its accuracy highly dependent on tumor sample availability, FFPE quality and sampling location. A blood‐only design that bypasses tumor requirement appears to be more convenient, and has been shown to achieve comparable accuracy with tumor‐guided approach in colorectal cancer when other epigenomic features were used together with mutations to identify ctDNA [36, 37].

## 5 Conclusions

In conclusion, we provided the somatic variant landscape of Vietnamese breast cancer women and established a personalized K‐Track^®^ assay to identify patients with residual cancer. Although the performance of the assay needs to be fully reported after completion of the study, this report suggests that K‐Track^®^ could be the affordable leading approach to empower precision oncology in Vietnam and possibly in other developing countries.

## Conflict of interest

V‐ANH, PLD, NTNT, MLN, NMN, DQN, Y‐TL, M‐DP, HG and LNT are current employees of Gene Solutions, Vietnam. The remaining authors declare no conflict of interest.

## Author contributions

STN, TVN, THP, TCD, DHP, DSN, DQN, Y‐TL, TTTD and DKT recruited patients and performed clinical analysis. V‐ANH, PLD, NTNT, MLN, NMN, M‐DP, H‐NN and HG processed samples and analyzed genetic data. LNT conceived and designed the project, analyzed data and wrote the manuscript. All authors contributed to the article and approved the submitted version.

## Supporting information


**Fig. S1.** The top 10 significantly mutated genes in each breast cancer subtype.
**Fig. S2.** Comparing mutation frequency with published datasets for each breast cancer subtype.
**Fig. S3.** Hotspot mutations in top mutated genes.
**Fig. S4.** Analytical performance of ctDNA detection assay.
**Table S1.** Patient demographics.
**Table S2.** List of 95 targeted genes.
**Table S3.** Actionable alterations and OncoKB™ therapeutic level of evidence.
**Table S4.** Frequency of hotspot mutations in top mutated genes among different cohorts.

**Table S5.** List of all somatic variants detected in FFPE and plasma samples.

## Data Availability

The genetic data that support the findings of this study are available in the supplementary material of this article.
