# Supplementary material for: Genetic landscape and personalized tracking of tumor mutations in Vietnamese women with breast cancer
Source: Mol Oncol. 2023 Jan 15;17(4):598–610. doi: 10.1002/1878-0261.13356 (PMC10061293; doi:10.1002/1878-0261.13356)
Supplement: Supplementary file 1 — Fig. S1. The top 10 significantly mutated genes in each breast cancer subtype. Fig. S2. Comparing mutation frequency with published datasets for each breast cancer subtype. Fig. S3. Hotspot mutations in top mutated genes. Fig. S4. Analytical performance of ctDNA detection assay. Table S1. Patient demographics. Table S2. List of 95 targeted genes. Table S3. Actionable alterations and OncoKB™ therapeutic level of evidence. Table S4. Frequency of hotspot mutations in top mutated genes among different cohorts. [file MOL2-17-598-s001.docx]

**Table S1. Patient demographics**

| Characteristic | N = 134 |
| --- | --- |
| Median age at diagnosis (range), year | 52 (25-78) |
| < 50, N (%) | 76 (56.7) |
| ≥ 50, N (%) | 58 (43.3) |
| Menopausal status, N (%) |  |
| Pre-menopause | 47 (35.1) |
| Peri-menopause | 16 (11.9) |
| Post-menopause | 71 (53.0) |
| Invasive carcinoma, N (%) |  |
| Ductal | 129 (96.3) |
| Lobular | 2 (1.5) |
| Not available | 3 (2.2) |
| Receptor status, N (%) |  |
| HR+ HER2- | 69 (51.5) |
| *Ki67 Low (< 10%)* | 37 (27.6) |
| *Ki67 High* | 32 (23.9) |
| HR+ HER2+ | 23 (17.2) |
| HR- HER2+ | 26 (19.4) |
| HR- HER2- | 16 (11.9) |
| Risk of recurrence classification, N (%) |  |
| HR+ HER2- | 69 (100) |
| *Low risk* | 37 (53.6) |
| *High risk* | 32 (46.4) |
| HR+ HER2+ | 23 (100) |
| *Low risk* | 5 (21.7) |
| *High risk* | 18 (78.3) |
| Size of tumor, mean (range), cm | 2.4 (0.4-10) |
| Number of tumor, mean (range) | 1 (1-3) |
| Clinical nodal status, N (%) |  |
| Negative | 52 (38.9) |
| Positive | 78 (58.2) |
| Not available | 4 (2.9) |
| Histological grade, N (%) |  |
| 1 | 3 (2.2) |
| 2 | 92 (68.7) |
| 3 | 25 (18.7) |
| Not available | 14 (10.4) |
| TNM stage, N (%) |  |
| I | 31 (23.1) |
| II | 69 (51.5) |
| III | 30 (22.4) |
| Not available | 4 (3.0) |

**Table S2. List of 95 targeted genes**

| *ACVR2A* | *CDKN2A* | *GATA3* | *NFE2L2* | *SETD2* |
| --- | --- | --- | --- | --- |
| *AFF3* | *CREBBP* | *GNAS* | *NOTCH1* | *SMAD4* |
| *AKT1* | *CTNNB1* | *GPC3* | *NOTCH2* | *SMARCA4* |
| *ALK* | *DDR2* | *GPHN* | *NRAS* | *SPOP* |
| *AMER1* | *DICER1* | *GRIN2A* | *NSD1* | *STAG2* |
| *APC* | *DNMT3A* | *HLA-A* | *NTRK3* | *STK11* |
| *AR* | *EGFR* | *HRAS* | *PDE4DIP* | *TBX3* |
| *ARID1A* | *EP300* | *IDH1* | *PIK3CA* | *TCF7L2* |
| *ARID1B* | *ERBB2* | *KDM6A* | *PIK3R1* | *TP53* |
| *ARID2* | *ERBB3* | *KEAP1* | *PREX2* | *TRRAP* |
| *ATM* | *ERBB4* | *KMT2A* | *PTEN* | *TSC1* |
| *ATR* | *ERCC2* | *KMT2C* | *PTPN13* | *TSC2* |
| *AXIN1* | *ESR1* | *KMT2D* | *PTPRB* | *TSHR* |
| *BCOR* | *FAT1* | *KRAS* | *PTPRT* | *ZFHX3* |
| *BRAF* | *FAT4* | *LRP1B* | *RAD51B* | *ZNF521* |
| *BRCA1* | *FBXW7* | *MAP3K1* | *RB1* |  |
| *BRCA2* | *FGFR3* | *MET* | *RBM10* |  |
| *CAMTA1* | *FHIT* | *NCOR1* | *RNF213* |  |
| *CASP8* | *FOXA1* | *NCOR2* | *RNF43* |  |
| *CDH1* | *FOXP1* | *NF1* | *RSPO2* |  |

**Table S3. Actionable alterations and OncoKB™ therapeutic level of evidence**

| Gene | Alterations | Drug | Level* |
| --- | --- | --- | --- |
| *BRAF* | V600E | Dabrafenib + Trametinib* | 1 |
| *ERBB2* | Amplification | Ado-Trastuzumab Emtansine, Lapatinib + Capecitabine/Letrozole, Margetuximab + Chemotherapy, Neratinib, Neratinib + Capecitabine, Trastuzumab, Trastuzumab +/- Pertuzumab + Chemotherapy, Trastuzumab + Tucatinib + Capecitabine, Trastuzumab Deruxtecan | 1 |
| *NTRK1/2/3* | Fusions | Entrectinib, Larotrectinib* | 1 |
| *PIK3CA* | C420R, E542K, E545A/D/G/K, H1047L/R/Y, Q546E/R | Alpelisib + Fulvestrant | 1 |
| *PIK3CA* | Oncogenic mutations | Alpelisib + Fulvestrant | 2 |
| *ERBB2* | Oncogenic Mutations | Neratinib | 3 |
| *AKT1* | E17K | AZD5363 | 3 |
| *ESR1* | Oncogenic Mutations | Fulvestrant | 3 |
| *BRCA1* | Oncogenic Mutations | Olaparib, Talazoparib | 3 |
| *BRCA2* | Oncogenic Mutations | Olaparib, Talazoparib | 3 |
| *NRG1* | Fusions | Zenocutuzumab* | 3 |
| *NTRK1/2/3* | Fusions | Repotrectinib* | 3 |
| *ARID1A* | Truncating Mutations | PLX2853, Tazemetostat* | 4 |
| *BRAF* | G464, G469A, G469R, G469V, K601, L597 | PLX8394* | 4 |
| *CDK12* | Truncating Mutations | Pembrolizumab, Nivolumab, Cemiplimab* | 4 |
| *CDKN2A* | Oncogenic Mutations | Palbociclib, Ribociclib, Abemaciclib* | 4 |
| *FGFR1/2/3* | Oncogenic Mutations | Debio1347, Infigratinib, Erdafitinib, AZD4547**^*^** | 4 |
| *KRAS* | Oncogenic Mutations | Trametinib, Binimetinib, Cobimetinib* | 4 |
| *MET* | Fusions | Crizotinib* | 4 |
| *MTOR* | Oncogenic Mutations | Everolimus, Temsirolimus* | 4 |
| *NF1* | Oncogenic Mutations | Cobimetinib, Trametinib* | 4 |
| *PTEN* | Oncogenic Mutations | GSK2636771, AZD8186* | 4 |
| *NTRK1* | G595R | Larotrectinib* | R1 |
| *NTRK1* | G595R | Entrectinib* | R2 |
| *NTRK3* | F617L, G623R, G696A | Larotrectinib* | R1 |

* Drugs for all solid tumors

Level 1: FDA-recognized biomarker for FDA-approved drugs

Level 2: Standard care biomarker recommended by professional guidelines for FDA-approved drugs

Level 3: Compelling clinical evidence supports the biomarker for a drug

Level 4: Compelling biological evidence supports the biomarker for a drug

Level R1: Standard care biomarker predictive of resistance to FDA-approved drugs

Level R2: Compelling clinical evidence supports the biomarker as being predictive of resistance to a drug

**Table S4. Frequency of hotspot mutations in top mutated genes among different cohorts**

| Gene | Nucleotide change | Amino acid change | TCGA-Caucasian (n=757) | METABRIC (n=2433) | TCGA-Asian (n=60) | Korea (n=187) | Malaysia (n=546) | Vietnam (n=134) |
| --- | --- | --- | --- | --- | --- | --- | --- | --- |
| *PIK3CA* | c.3140 A>G c.3140 A>T | p.H1047R p.H1047L | 74 (9.8) | 465 (19.1) | 10 (16.7) | 27 (14.4) | 70 (12.8) | 31 (27.6) |
|  | c.1633 G>A | p.E545K | 39 (5.2) | 291 (11.9) | 3 (5.0) | 11 (5.9) | 35 (6.4) | 6 (4.5) |
|  | c.1624 G>A | p.E542K | 26 (3.4) |  | 3 (5.0) | 3 (1.6) | 11 (2.0) | 5 (3.7) |
|  | c.1035 T>A | p.N345K | 14 (1.8) | 64 (2.6) | NA | 3 (1.6) | 9 (1.6) | 2 (1.5) |
| *TP53* | c.742 C>T c.743 G>A | p.R248W p.R248Q | 3 (0.4) | 60 (2.5) | 1 (1.7) | 6 (3.2) | 14 (2.6) | 4 (2.9) |
|  | c.711G>A | p.M237I | NA | NA | NA | 1 (0.5) | NA | 3 (2.2) |
| *GATA3* | c.1223_1224insT | p.P409Afs*99 | 6 (0.8) | NA | 1 (1.7) | 7 (3.7) | 12 (2.2) | 5 (3.7) |
| *AKT1* | c.49G>A | p.E17K | 14 (1.8) | 80 (3.3) | 2 (3.3) | 5 (2.7) | NA | 4 (3.0) |

**
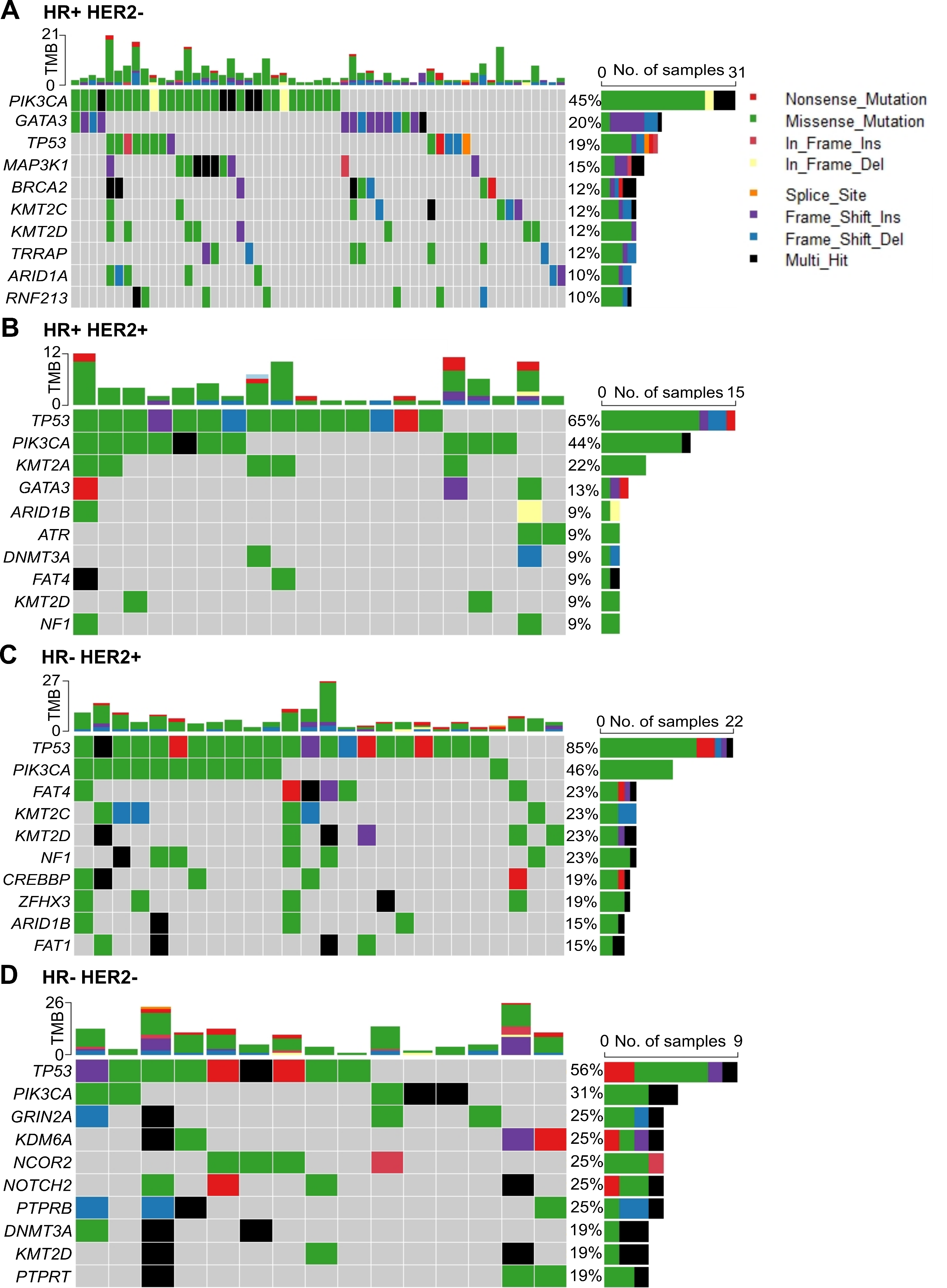
**

**Figure S1. The top 10 significantly mutated genes in each breast cancer subtype.** *PIK3CA* and *TP53* were among the top three mutated genes in all subtypes. Majority of mutations were missense.

**
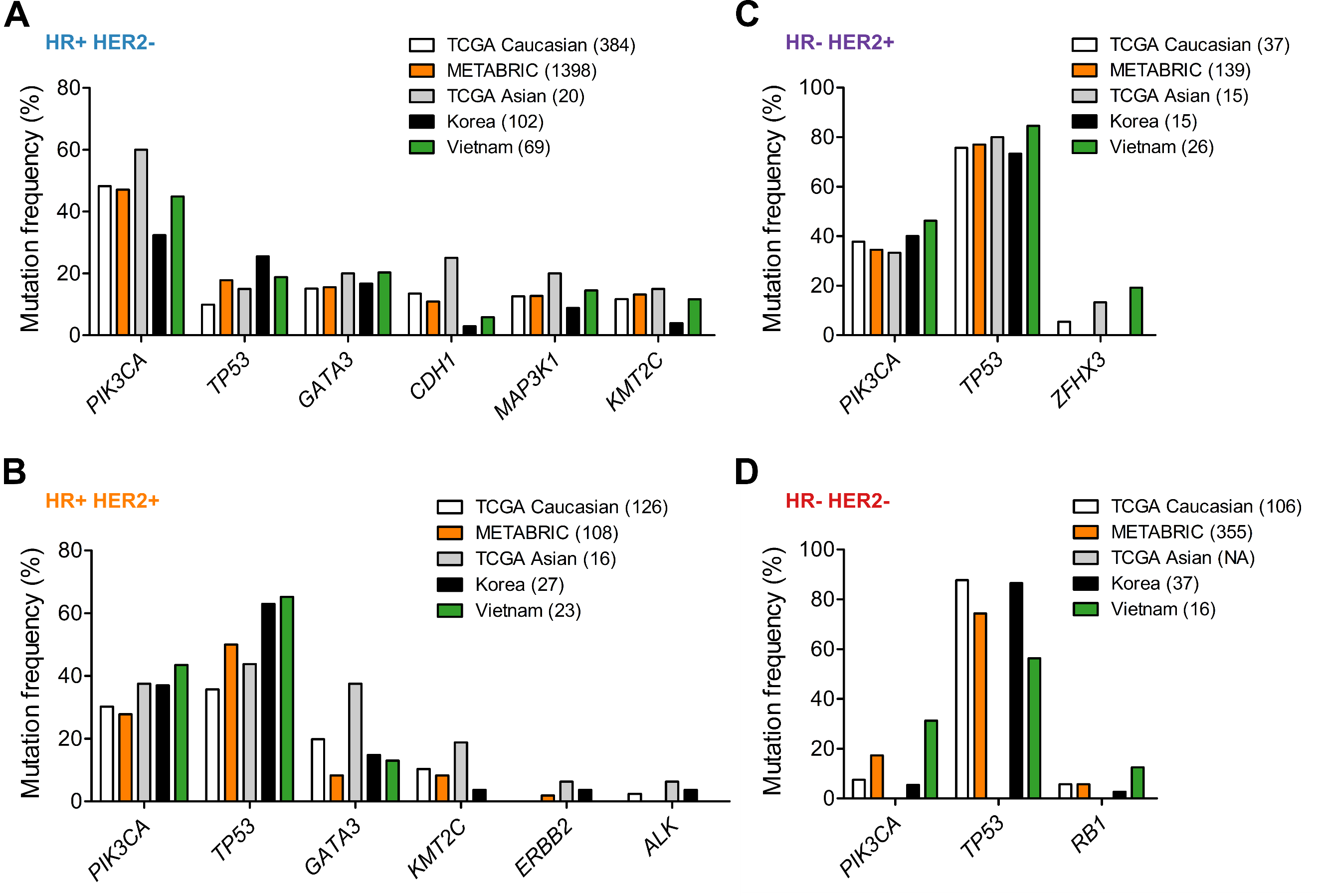
**

**Figure S2. Comparing mutation frequency with published datasets for each breast cancer subtype.** Frequency of top highly mutated genes in each subtype was compared between our cohort and published datasets of Caucasian and Asian cohorts. The main observation was higher *ZFHX3* mutation rate in our HR- HER2+ group, higher *PIK3CA* while lower *TP53* mutation rate in our HR- HER2- group compared to other cohorts.

**
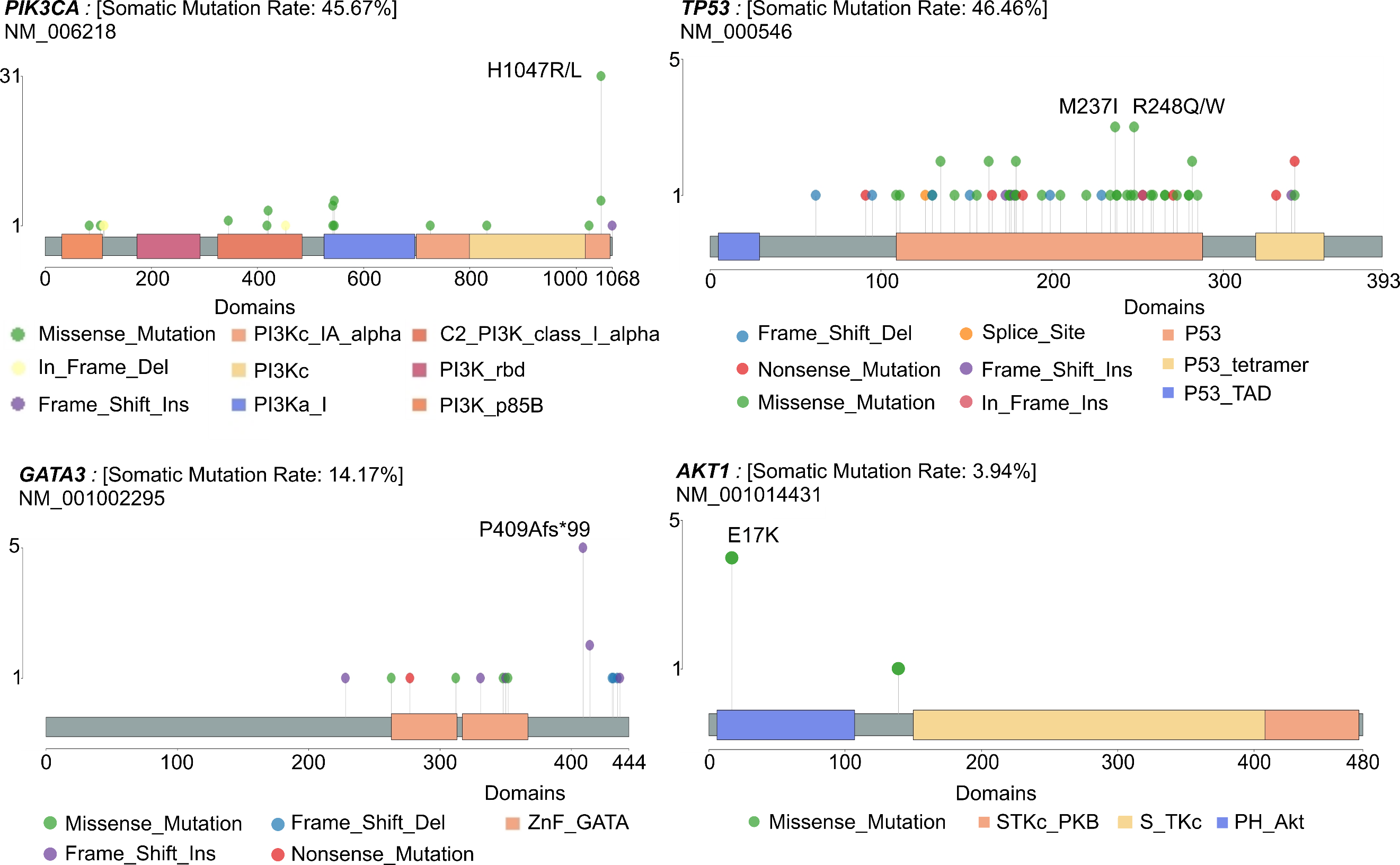
**

**Figure S3. Hotspot mutations in top mutated genes.** Lollipop plots displaying the mutation distribution and protein domains for PIK3CA, TP53, KMT2C and APC. *PIK3CA* H1047R/L, *GATA3* P409Afs*99 and *AKT1* E17K could be the recurrent mutations in the Vietnamese breast cancer women.

**
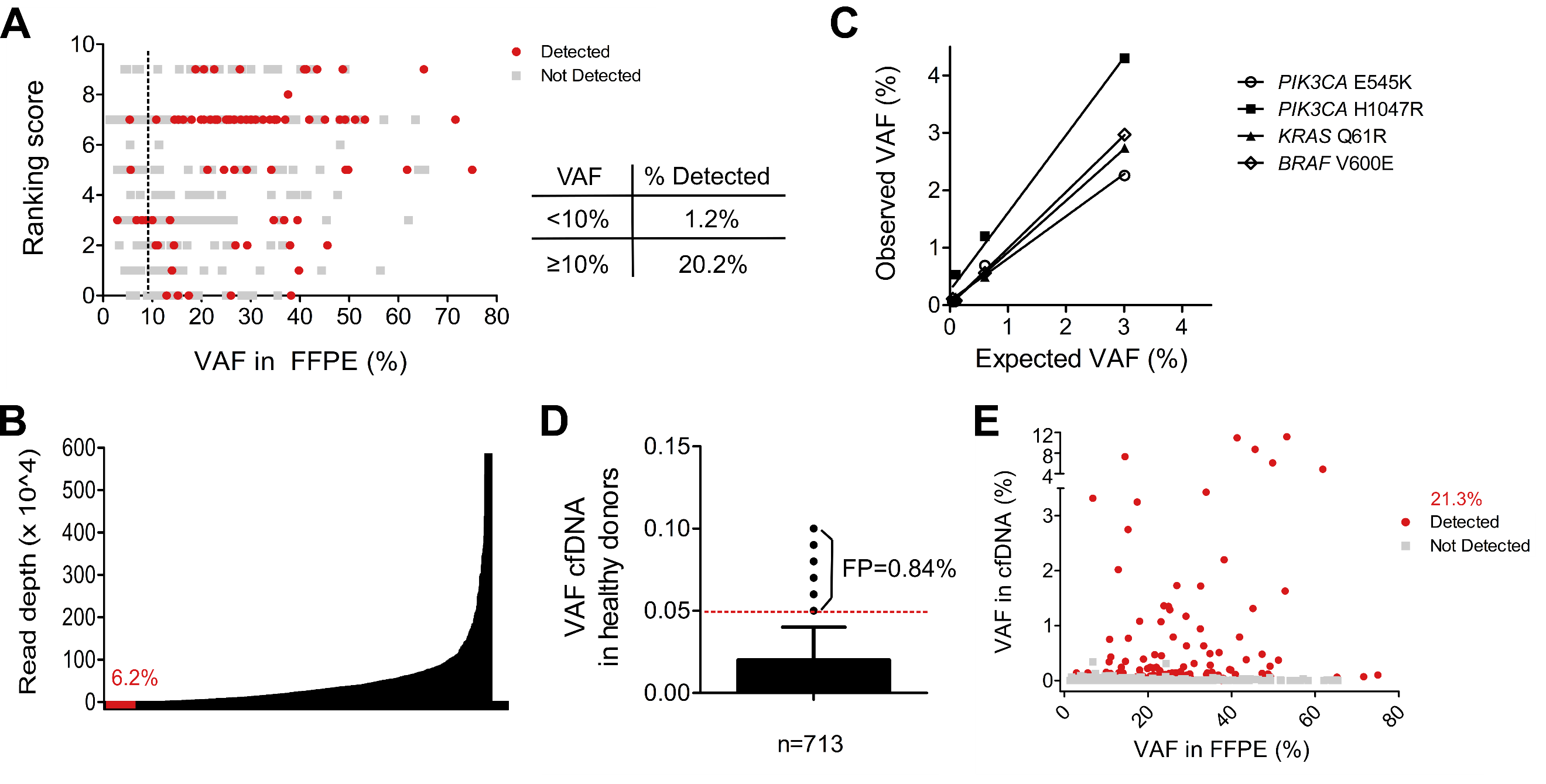
**

**Figure S4. Analytical performance of ctDNA detection assay. (A)** VAF of a mutation in FFPE was the more determining factor than ranking score to affect its detection rate in plasma. Red dots = mutation detected in the plasma; grey dots = mutations not detected in the plasma. **(B)** The distribution of read depth per amplicon in all analyzed plasma samples. Amplicons with sequencing coverage <10,000X (marked red) were excluded from downstream analysis. **(C)** Graph of the titration series for *PIK3CA* E545K, *PIK3CA* H1047R, *KRAS* Q61R, and *BRAF* V600E to determine limit of detection at 0.05%. **(D)** Target-level specificity of the K-Track® assay was > 99% with the false-positive signals from healthy plasma (n=100 samples, 713 amplicons) at 0.84%. **(E)** 21.3% of tracked FFPE mutations were detected in cfDNA. There was no correlation between VAF of a mutation in FFPE and its detected VAF in cfDNA.
